# Risk factors for signs of tail biting in intact-tailed weaner pigs: A cross-sectional study

**DOI:** 10.1017/awf.2026.10076

**Published:** 2026-03-27

**Authors:** Camilla Munsterhjelm, Miina Tuominen-Brinkas, Hilkka Koskikallio, Mari Heinonen, Kristina Ahlqvist, Anna Valros

**Affiliations:** Production Animal Medicine, University of Helsinki, Finland, Finland

**Keywords:** Animal welfare, cannibalism, post-weaning, *Sus scrofa domesticus*, tail lesion, weaner

## Abstract

Tail biting in growing pigs is both a sign and cause of impaired welfare. On one-day visits to ten Finnish farms, we assessed weaned pigs between approximately 4 to 10 weeks of age. On each farm, tail health was assessed at the pen level in 2–15 rooms (n = 81) selected randomly within age strata (n = 3), and on individual level in one high-prevalence pen per age category (n = 37 pens). Risk factors for tail biting were assessed in 2–3 age-representative rooms per farm according to 57 resource- and animal-based questions on environment, hygiene, health, feeding and enrichment. Tail health data were reduced into three pen- and three individual-level principal components. Component scores were used as outcome variables when establishing effects of risk factors on tail health using regression tree analysis and mixed modelling. Tail health deteriorated over the weaner period, especially from the first to the second third. Besides age, risk factors for impaired tail health consisted predominantly of shortcomings related to basic needs, such as adequate resting areas and enrichment. In the individual-level data, different risk factors could be associated with different types of tail lesions in problem pens, suggesting distinct aetiologies. In conclusion, improvements to the early environment in the farms studied here may have a significant positive impact on the well-being of pigs, not only post-weaning, but potentially permanently if experiencing less tail biting in early life leads to prevention later.

## Introduction

Tail biting is a major cause of animal suffering and economic losses in intensive pig production. The behaviour is considered a reaction to stress or impaired welfare (Schrøder-Petersen & Simonsen 2001; Widowski 2002), the causes of which are often associated with shortcomings in housing conditions or husbandry (Henry *et al.* 2021). Determining the causes of tail biting in any given situation is complicated by potential interactions with internal factors, such as health status, genetics, stress physiology (Henry *et al.* 2021) and potentially also gut microbiota (Brunberg *et al.* 2016; Rabhi *et al.* 2020; König *et al.* 2024). One internal factor which can be controlled is previous experience of tail biting which is recognised, anecdotally, as lowering the threshold for later outbreaks (Valros *et al.* 2016). This observation appears to reflect the notion that an individual with a previous tail lesion is at increased risk of being bitten (Lahrmann *et al.* 2018; Håkansson *et al.* 2020; Munsterhjelm *et al.* 2025). The interval between primary and secondary victimisation may extend up to a few months (Ursinus *et al.* 2014). An outbreak of tail biting may be difficult to control due to its escalating nature. According to Niemi *et al.* (2021), the proliferation of tail lesions in a room can be modelled as a dynamic process, in many ways resembling the spreading of a pathogen. The transmission of tail-biting behaviour has been suggested as occurring as a result of imitation (Blackshaw 1981), or the attractiveness of blood (Fraser 1987).

Previous research on tail biting has focused on the finishing phase, probably because the most severe lesions and economic consequences will be more evident in heavier animals. However, an improved overall outcome would be dependent upon the prevention of early outbreaks of such behaviour. The onset of tail biting tends to be seen in the weaner period (Valros *et al.* 2020, 2021) or perhaps even earlier, in the suckling stage (Håkansson *et al.* 2020). Risk factors for tail biting most likely differ for weaner pigs compared to fattening pigs due to the significant differences in physiology. For example, weaning causes negative energy balance, increasing animals vulnerability to the cold and leading to suboptimal feeding for the first week (Le Dividich & Herpin 1994). To facilitate the understanding of tail biting in weaner pigs, this study aimed to: (1) describe the quality and occurrence of indicators of tail biting and prevalence of known risk factors in weaner pigs on commercial farms; and (2) establish associations between these indicators and risk factors.

## Materials and methods

### Ethical statement

The University of Helsinki Viikki Campus Research Ethics Committee gave ethical approval to this study (Statement 2/2022).

### Study farms

Ten farms showing historically different levels of tail biting in the weaner period were selected from the three largest slaughterhouse companies in Finland, thereby ensuring all genetic breeds used in the country were included (DanAvl, Topigs Norsvin and Figen). No pigs were tail docked, but all males were castrated and for all animals weaning age was approximately four weeks. Piglets were born on the farm with the exception of farm 2 (where animals from farm 1 were raised). The animals were typically transported for up to 30 min, and litters mixed at introduction to the weaner rooms. Prior to the start of the study the mean (± SD) weight at arrival and departure from the weaner pig section were 7.6 (± 0.7) and 34.6 (± 3.2) kg, respectively.

The smallest farm (farm 5) practiced continuous throughput in a system with one room for small and one for large weaner pigs. Although the other farms were built for all-in, all-out production by room, this was only strictly adhered to on two farms, with the others reporting that some animals were moved between rooms to maximise throughput. Buildings were insulated and heated, and all farms featured partly slatted pen floors. Two-climate pens were present on all farms except farm 5. Five of the farms provided liquid feed from a long trough, four provided dry feed from a feeder, and the last farm (farm 5) fed liquid feed to the small and dry feed to the larger weaner pigs. The minimum regime of enrichment was set by the national interpretation of relevant EU legislation, requiring either continuous or twice daily provision of a rootable material in an amount that animals can move into a small pile; with an addition of toys in the latter alternative (Finnish Food Authority 2019). The choice of rootable material was straw or hay, typically in combination with sawdust or peat on nine farms while one farm used sawdust only. Additional enrichment objects, typically wood blocks, plastic discs or rubber nipples, were provided on eight farms. Further production characteristics can be found in Table 1.

**Table 1.** Characteristics of weaner pig housing on 10 farms. The data describe the sampled rooms, but are representative of the farm

Farm	Weaner pigs^a^ (n)	Pens per room (n)	Group size (pigs, n)	Room size		
				Average (pigs, n)	CV	Space allowance m^2^ per pig
1	9,000–12,000	16	14–16	240	6%	0.33–0.42
2	4,000–8,000	16	30–60^b^	495	33%	0.42–0.54
3	4,000–8,000	20	25–30	470	19%	0.37–0.46
4	≤2,000	12	10–15	146	18%	0.45–0.48
5	≤2,000	16	12	180	9%	0.47–0.60
6	4,000–8,000	16	22–25	354	15%	0.41–0.57
7	4,000–8,000	12	23–28	292	8%	0.41–0.50
8	4,000–8,000	20	17–19	367	16%	0.41–0.48
9	9,000–12,000	28	23	644	0%	0.39–0.44
10	9,000–12,000	16	12–13	205	3%	0.48–0.51

CV=coefficient of variation between rooms included in the sample

a Average number of weaner pigs over 12 months before the visit; categorized for anonymity; source: national herd health database Sikava

b Pens were overfilled at arrival and thinned out later

### Data collection

A tail biting Risk Factor Questionnaire (RQ) including 57 questions was prepared based on literature and practical experience of pig production in the country. Both resource- and animal-based questions were included, with the majority describing the current situation at time of completion in the pens. Thus data regarding usage of enrichment materials, for example, were based not on the opinion of a stockperson, but on actual observations within the pens. Most data were collected in predefined categories, with an increased category number denoting an increasing risk. The complete questionnaire can be found in Table S1 (see Supplementary material) and is summarised in Table 2. In the following text, questions and answers are referred to by their number following the letter Q and C, respectively. Q10C2 is, for example, short for question 10, answer category 2.

**Table 2.** Summary of the Risk Factor Questionnaire, used to collect information on risks for tail biting in weaner pigs on 10 farms. Details are given in Table S1 (Supplementary material)

Group of questions	Total no of questions	Questions, animal-based	Questions, resource-based
Air quality, noise, light, draught, mist system	7		34–40
Availability, quality, diversity and usage of bedding, rooting material and other enrichment items	7		22–28
Behaviour and lesions (including tails) as an indicator of restlessness or shortage of resources	4	21,45,56,57	
Behaviour as indicator of temperature (panting, shivering)	2	32,33	
Demographics and general: Weaner capacity, group size, pens and sick pens per room, pen uniformity, age and weight category	7	2,3	1, 4–7
Feeding system, feed, feeding-related hygiene, feed availability, pigs with empty bellies	8^a^	20^a^, 46	15,16,41–44
Group-level resting behaviour as an indicator of temperature and availability of roofed, clean, solid and/or bedded area	2	12,31	
Space allowance, floor type, activity areas, quality of resting area	8	13,14,48	8–11, 29
Runts and size uniformity in pens	2	54,55	
Symptoms: diarrhoea, anaemia	2	49,53	
Symptoms: pruritus	1	52	
Symptoms: respiratory	2	50,51	
Temperature, lying area	1		30
Water availability and hygiene	5^a^		17–20^a^, 47

a Q20 “Resource accessibility for unprivileged individuals” is evaluated in terms of both feed and water access

Each farm was visited once for assessment of tail status and risk factors. A representative of the farm was questioned on farm characteristics and husbandry routines. Weaner pig capacity and room size were the only interview-obtained information used in statistical analyses, with all other data collected by the researchers. Data collection in the barn consisted of three stages: (1) observation of tail status at pen level; (2) collection of RQ data; and (3) detailed assessment of tail damage at an individual level. On each farm, pen-level tail status was observed in a sufficient number of rooms to reach an approximate total of 200 pens. The rooms were randomly selected within strata representing three age categories, which are represented by the ordinal number of the week in the section (weeks 1–2, 3–4 and 5–6 in the weaner section). Sample sizes for all collected data are shown in Table 3. Assessors entered the pen but did not restrain or touch the animals. For each pen, the point prevalence of healed, shortened tails, fresh tail lesions and hanging tails were categorised as no, up to 10%, or exceeding 10% of animals (Table 4). The threshold for a fresh lesion was obvious redness, i.e. penetration of the skin was not required. A 10% prevalence separated low- and high-prevalence pens, whereas one case was required for a pen to be considered affected by tail biting. These limits were selected based on literature: applied thresholds for a pen-level tail-biting event have included tail damage both in 10% of animals (Wedin *et al.* 2018; D’Eath *et al.* 2021; Drexl *et al.* 2024); as well as just one case (Domun *et al.* 2019; Larsen *et al.* 2019).

**Table 3.** Sample sizes for the Risk Factor Questionnaire (RQ, rooms), pen-level tail health (T_pen_, rooms with all pens assessed) and individual-level tail health data (T_ind_, pens/ individuals) in three age categories (weeks in weaner pig section); as well as percentage of weaner pig capacity on the farm included in the samples

Farm	% of capacity assessed^1^			1–2 Weeks			3–4 Weeks			5–6 Weeks		
	RQ	T_pen_	T_ind_	RQ	T_pen_	T_ind_	RQ	T_pen_	T_ind_	RQ	T_pen_	T_ind_
1	8%	33%	0. %	1	3	1/12	1	5	2/27	1	5	2/29
2	21%	75%	1.9%	1	3	1/25	1	3	2/54	1	5	2/58
3	32%	85%	2.6%	1	2	1/29	1	3	3/68	1	3	1/21
4	24%	48%	0.6%	1	2	0	1	1	1/11	1	3	0
5	55%	55%	13.1%	0	0	2/67	1	1	0	1	1	2/24
6	19%	43%	1.1%	1	2	0	1	2	2/55	2	5	1/26
7	14%	41%	1.2%	1	1	1/33	1	5	1/20	1	3	1/23
8	20%	39%	1.1%	1	2	1/19	2	4	2/43	0	0	0
9	21%	14%	0.9%	1	1	0	1	1	4/84	0	0	0
10	6%	28%	0.4%	1	7	0	1	5	4/49	1	3	0

1 Percentage of capacity (weaner pig rooms for RQ, weaner pig pens for T_pen_ and weaner pigs for T_ind_) included in the sample.

**Table 4.** Process of pen-level tail health evaluation in weaner pigs. The steps are completed in each pen in the given order

1) Standing inside the pen the number of pigs with the following findings is determined
Tail lesion: change indicative of biting, at least reddening of the skin
Hanging tail: un-curled and kept below the spine level
Healed, shortened tail: visible shortening, but no recent lesion (no reddening, wound or crust)
2) The pen is assigned to the most severe category (highest on the list):
Lesion in > 10%^a^ of animals
Lesion in at least one^b^, altogether in ≤ 10% ^a^ of animals
Hanging tail in > 10% ^a^ of animals
Hanging tail in at least one^b^, altogether in ≤ 10% ^a^ of animals
No signs of recent tail biting
3) The pen is assigned to either category
Healed, shortened tail in > 10% ^a^ of animals
Healed, shortened tail in ≤ 10% ^a^ of animals

Threshold determined according to ^a^D’Eath *et al.* (2018), Wedin *et al.* (2018), Drexl *et al.* (2024); or ^b^Domun *et al.* (2019), Larsen *et al.* (2019).

RQ data were collected as shown in Table S1 (see Supplementary material) in three rooms per farm, representing the three age categories. Rooms with a relatively high prevalence of lesions were selected. All pens, including sick pens, were included in the assessment. If animals within an age category were housed in vastly different types of rooms, typically due to older and newer sections present on the farm, separate RQs were completed. This was true for age category weeks 5–6 on farm 6 and weeks 3–4 on farm 8.

In the final phase of data collection tail damage was assessed in detail according to Table 5 in all individuals in problem pens. The aim was to include four of the most severely affected pens on each farm, including all age categories if possible. Board restraint was used and tails touched, as necessary for close inspection.

**Table 5.** Tail health assessment chart for individual data collection in weaner pigs

Variable	Category	Definition
Tail Posture	Not hanging	The tail is curled, and if not, it is kept above a horizontal line originating at its insertion
	Hanging	The tail is not curled and kept below a horizontal line originating at its insertion
Tail Shortening	No	The tail appears not to be shortened. The tip is flattened.
	Shortened	The tail is visibly shortened, but at least 7.5 cm long: the end is scarred and/or a part is missing and/or the physiological flattening or the tip is replaced by thickening.
	Stump	The tail is less than 7.5 cm long.
Lesion Type^1^	No lesion	There are no fresh or healing lesions, but there may be scar tissue
	Lesion < 5 mm	The skin is penetrated by a fresh or healing lesion smaller than 0.5 cm in size^2^
	Lesion 0,5–2 cm	The skin is penetrated by a fresh or healing lesion 0.5 to 2 cm in size^2^
	Lesion > 2 cm	The skin is penetrated by a fresh or healing lesion larger than^2^ 2 cm
Lesion Freshness^3^	No lesion	There is no evidence of skin-penetrating lesions, current or healed: fresh or healing lesions, or scar tissue
	Fresh or acute	There is blood or a red-coloured crust, and/or tissue underlying the skin is visible. The tissue is moist and may be red, grey, or dark.
	Old crust	There is a dark-coloured crust
	Healed lesion	There is scar tissue: skin-like or light pink with a dry surface

1 The tail is categorised according to the most severe lesion present.

2 The size is the diameter of an area or the length of a linear lesion.

3 Recorded only for skin-penetrating lesions, acute or healed. All Freshness categories visible are recorded irrespectively of their size.

### Data processing and univariate statistical analyses

SPSS 29.0 was used for statistical analyses. RQ variables describing environmental dimensions were expressed in more than one unit, if considered biologically relevant: Q30 *Resting area temperature* and Q19 *Water flow* were expressed as mean and SD, min and max in the room, with the new variables denoted by subscripts (e.g. Q30_avg_). Q8 *Space allowance*, Q9 *Solid floor area* and Q29 *Roofed area* were expressed as absolute (m^2^ per pen, subscript_pen) and relative (m^2^ per animal, subscript_ind). With these additions the total number of RQ variables rose to 67. RQ data, which were collected in one representative room per age category on each farm, were replicated within age category. On farms 6 and 8, where animals within an age category were kept in two vastly different types of rooms, replication was performed within room type. Pen-level tail status data were expressed on room level as the percentage of pens in each category given under (2) and (3) in Table 4 (n = 7 variables). Age category effects on tail health variables were analysed using the Independent-Samples Kruskal-Wallis test.

### Multivariate statistical analyses

#### Principal Component Analysis

Principal components were determined in both room- and individual-level tail status data in order to reduce data dimensions, identify latent types of tail damage type and occurrence across the data, and obtain scores for each of these types to use as outcomes in further analyses. Room-level data were continuous and thus analysed using PCA in the Factor Analysis feature. The variable ‘Shortened, healed tail’ was removed due to low extraction communality (score < 0.2). Rotations failed to increase the percentage of total variance explained or facilitate the interpretation of results and thus were not applied. For individual-level analyses, dichotomous dummy variables were created, indicating the presence or absence of each category for each variable. The original three variables (shortening, lesion type and lesion freshness) were thereby increased to a total of ten dummy variables, which were reduced into three principal components using the CATPCA feature with varimax rotation. Farm effects on component scores were tested using one-way ANOVA.

#### Regression Tree Analysis and linear mixed modelling

Regression Tree Analysis (RTA) and linear mixed regression (LMR) were used to investigate effects of risk factors (RQ items) on room-level patterns of tail lesion occurrence or individual-level tail status. PCA scores were used as outcome variables. As the scores are based on prevalence or occurrence values multiplied by the variable load, a high PCA score indicates that the type of tail-biting occurrence of damage in question was prevalent in relation to the whole sample of rooms (room-level outcome variables) or to the sampled problem pens (individual-level outcome variables). RQ data were used as predictors after removing Q17, Q22, Q32, Q33, Q35, Q45 and Q52 due to a lack of variability; as well as Q31 due to 19/81 missing values (animals could not be observed lying). Sex and room-level PCA scores as a measure of the population tail status were included as additional predictors in individual-level analyses.

RTA was the preferred method of analysis for the present data due to its ability to negotiate collinearity, a problem inherent in regression analysis with correlating predictors. RTA is well suited for analysis of multifactorial phenomena, such as effects of a multitude of risk factors on tail biting, as can be seen in EFSA (2014) and Scollo *et al.* (2017). RTA is based on repeated splitting of cases into subgroups or nodes, which are homogeneous in terms of tail status (PCA scores). The result can be presented as an easily interpreted tree showing the most important predictors, their effects and hierarchy. The Decision Tree package with a CRT algorithm was applied. Model accuracy was determined using 25-fold cross-validation for room-level data with the minimum number of cases for a child node set to 8; for individual-level data the cross validation was 10-fold and minimum child node set to 50. Both procedures limit tree growth and thereby overfitting of the data.

In cases where the RTA did not function satisfactorily, LMR using the Generalised Linear Model package was applied as a secondary analytic strategy. The LOWPREV_ROOM_ PCA score was log-transformed to achieve normality of residuals. Clustering of the data was accounted for by testing farm (n = 10) and room (n = 81); or farm and pen (n = 35), as random intercepts in room- and individual-level analyses, respectively. Age category (n = 3) was forced into all models as a fixed effect. Eligible predictors were identified in the RQ dataset based on significant univariate associations (results not shown). Inter-predictor correlation was common, raising concerns of multicollinearity which were mitigated by taking care to test correlating variables separately. For predictors considered to have an age-dependent effect, such as temperatures and dimensions of resources, interactions with age category were tested. Each model was approved upon determination of homoscedasticity and normality of residuals. *Post hoc* pair-wise comparisons with a least-significant difference adjustment were performed for categorical predictors with a significant main effect. Associations between risk factors and HEALED_IND_ were not established, as timing of tail injuries was unknown and the effect of the collected risk factors thus dubious.

## Results

### Descriptive and univariate results

**Figure 1.**
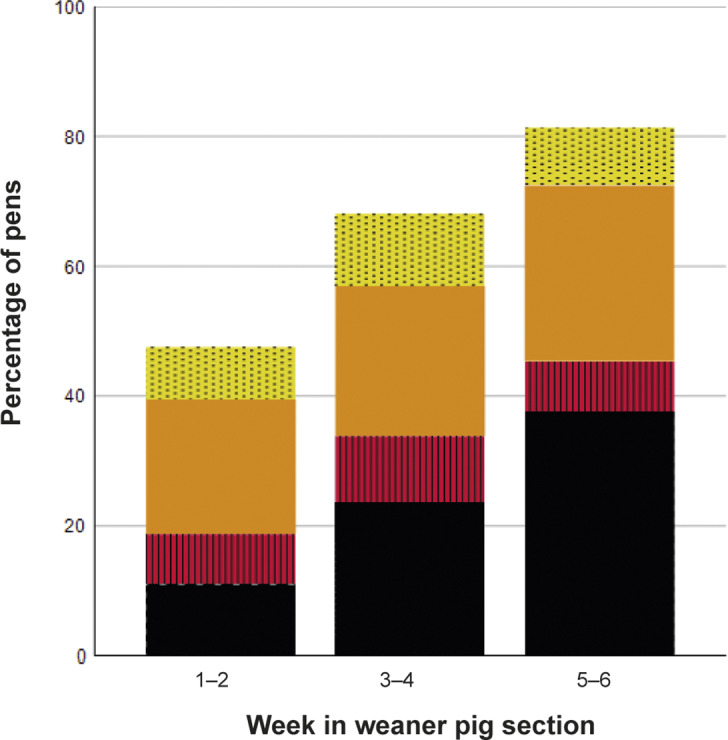
Tail health according to age in weaner pigs in 1,217 pens in 81 rooms on ten farms as average percentage of pens in each tail health category in the pooled data. Categorisation is based on the most severe tail findings, including lesion in > 10% of pigs in pen (black), lesion in ≤ 10% (red, striped), hanging tail in > 10% (orange) and hanging tail in ≤ 10% of pigs in pen (yellow, dotted).

Room-level tail status is detailed in Table 6 and Figure 1, and a farm-wise summary is given in Table S2 (see Supplementary material). Tail health in the pooled 81 rooms deteriorated with increasing age. Age category affected the percentage of pens with a tail lesion in > 10% of animals (*P* = 0.002) and the percentage of pens with no signs of recent tail biting (*P* < 0.001), with a tendency for hanging tails in > 10% of animals (*P* = 0.082, Kruskal-Wallis for all analyses, n = 81 rooms; Table 6). The median across rooms was equal for the second and final thirds, however, in the last third a subset of rooms reached very high proportions of pens with lesions. The type of tail lesions in problem pens (n = 35) differed greatly between farms (Table S2; Supplementary material).

**Table 6.** Age (week in weaner pig section) effects on room-level prevalence (median [min-max]) of pen-level measures of tail health in weaner pigs in 1,217 pens in 81 rooms on 10 farms

	1–2 week^e^ (n = 23)	3–4 week^e^ (n = 29)	5–6 week^e^ (n = 29)
Lesion^a^ in >10% of animals in the pen	10.0%^A^ (0.0–43.8)	25.0%^B^ (0.0–62.5)	26.7%^B^ (0.0–93.8)
Lesion^a^ in at least one, altogether ≤10 % of animals in the pen	6.3% (0.0–43.8)	6.7% (0.0–50.0)	6.7% (0.0–25.0)
Hanging tail^b^ in >10% of animals in the pen	12.5% (0.0–83.3)	25.0% (0.0–53.3)	21.1% (0.0–66.7)
Hanging tail^b^ in at least one, altogether ≤10% of animals in the pen	6.7% (0.0–35.7)	8.3% (0.0–50.0)	6.3% (0.0–50.0)
Healed, shortened tail^c^ in >10% of animals in the pen	0.0% (0.0–60.0)	0.0% (0.0–84.6)	0.0% (0.0–83.3)
Pens with healthy tails only^d^	50.0%^A-^ (7.1–100)	20.0%^B^ (0.0–93.3)	23.1%^B^ (0.0–81.3)

a A “lesion” was any sign of tail injury visible without touching the tail, standing in the pen. Skin penetration was not required.

b The tail is kept below a horizontal line at back level

c Tail visibly shortened

d Pens not in any of the other categories

e Week in the weaner pig section. Values with different superscript letters differ significantly (*P* ≤ 0.03) in the Kruskal-Wallis test.

Only a summarised version of the RQ results (n = 32 rooms) will be provided here, as the complete data are shown in the Table S1(b) and (c; see Supplementary material). Risk factors occurring with low and high frequency were identified according to the percentage of rooms in category 0 and in the highest category, as answer categories were defined to represent increasing tail-biting risk. As such, the most prevalent risk factors, true for all rooms in the data, were absence of a mist system (Q35) and no possibility for all animals to use enrichment simultaneously (Q22). Other highly prevalent risk factors included: Q12C3 *> 50% of pigs are resting at another location than the resting area* which was observed in 41% of rooms; Q23C3 *Rooting material is not available continuously* in 36%; Q28C3 *No bedding in > 80% of pens* in 36%; and Q55C3 *Clearly heterogenous size of pigs in > 50% of pens* in 33% of rooms. Factors with all rooms on the lowest risk level included: Q17 *Any water nipples in the lying area*; Q32 *Panting*; and Q33 *Shivering*; none of which were observed. Other low-prevalence risk factors included: Q6 *All pens are of equal size* which was true for 94% of rooms; Q26 *Bedding and enrichment materials are not dusty, mouldy, dirty etc* (93%); Q37 *The air is not perceived humid* (94%); Q47 *≤ 10% of nipples are dirty* (90%); Q52 *No scratching* (97%); and Q53 *No pale pigs* in 87% of rooms.

### Latent types of signs of tail biting

**Figure 2.**
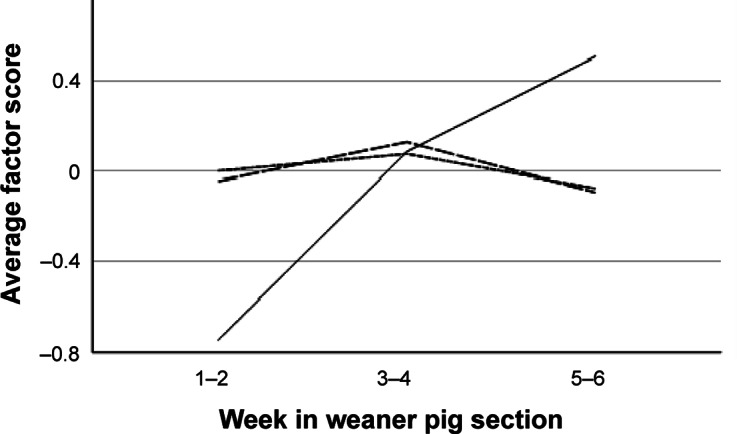
Average scores for principal components describing room-level patterns of occurrence of tail-biting signs in weaner pigs in 81 rooms on ten farms. The factors are “Recent high-prevalence tail biting” (HIGHPREV_ROOM_, solid line), “Recent low-frequency tail biting” (LOWPREV_ROOM_, long dash) and “Underlying tail biting” (UNDERLYING_ROOM_, short dash).

The three principal components extracted from room-level data (n = 81) explained a total of 84.5% of variance (33.6% + 27.9% + 23.0% for components 1–3, respectively). The first component was named “Recent high-prevalence tail biting” (HIGHPREV_ROOM_) as it was positively loaded by the percentage of pens with > 10% tail lesions (load 0.75) and > 10% hanging tails (0.45), and negatively by the percentage of pens with healthy tails (–0.95). The second component was named “Underlying tail biting” (UNDERLYING_ROOM_) as it was positively loaded by the percentages of pens with ≤ 10% (0.68) and > 10% of hanging tails (0.71). The third component included tail lesions in ≤ 10% (0.88) and hanging tails in ≤ 10% (0.46) and was named “Recent low-frequency tail biting” (LOWPREV_ROOM_). Average factor scores according to age category are given in Figure 2. All factor scores differed by farm (farm effect; *P* ≤ 0.005, n = 81, one-way ANOVA). Only HIGHPREV_ROOM_ was affected by age category (*P* < 0.001, Kruskal-Wallis), with a lower average score in weeks 1–2 as compared to weeks 3–4 and 5–6 (*P* < 0.01).

**Figure 3.**
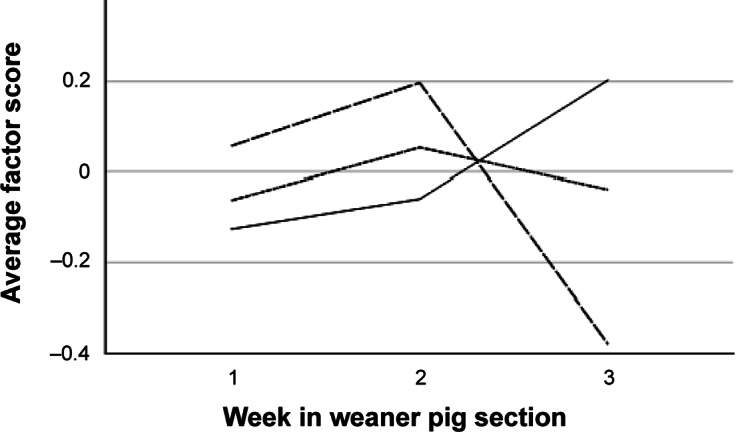
Average scores for principal components describing latent types of tail damage in weaner pigs (n = 777) in pens with high prevalence of tail lesions on ten farms. The factors are “Acute severe injury” (SEVERE_IND_, solid line), “Relatively fresh tail injury” (FRESH_IND_, long dash) and “Healed, shortened tail” (HEALED_IND_, short dash).

Three components explaining a total of 66.2% of variance in the data were extracted from individual-level data in pens with high tail-lesion prevalence (n = 777; for descriptive data, see Table S3; Supplementary material). The first component was named “Relatively fresh tail injury” (FRESH_IND_, explaining 24.1% of variance) as it was negatively loaded by Lesion type “no lesion” (–0.87) and Freshness categories “crust” (0.77), “scar” (–0.60) and “fresh/acute” (0.53). The second component was named “Acute severe injury” (SEVERE_IND_, 21.6%) as it was positively loaded by Lesion types “> 2 cm” (0.82) and “0.5–2 cm” (–0.68), Shortening category “stump” (0.63) and Freshness “fresh/acute” (0.61). The third component was positively loaded by Tail shortening “Shortened” (0.90) and “not shortened” (–0.85), as well as Freshness “healed” (0.47). It was named “Healed, shortened tail” (HEALED_IND_, 20.4%). Average factor scores according to age category are given in Figure 3. All factor scores were affected by age category and farm (*P* < 0.001, n = 777, one-way ANOVA).

### Effects of risk factors on room-level tail status

#### HIGHPREV_ROOM_

**Figure 4.**
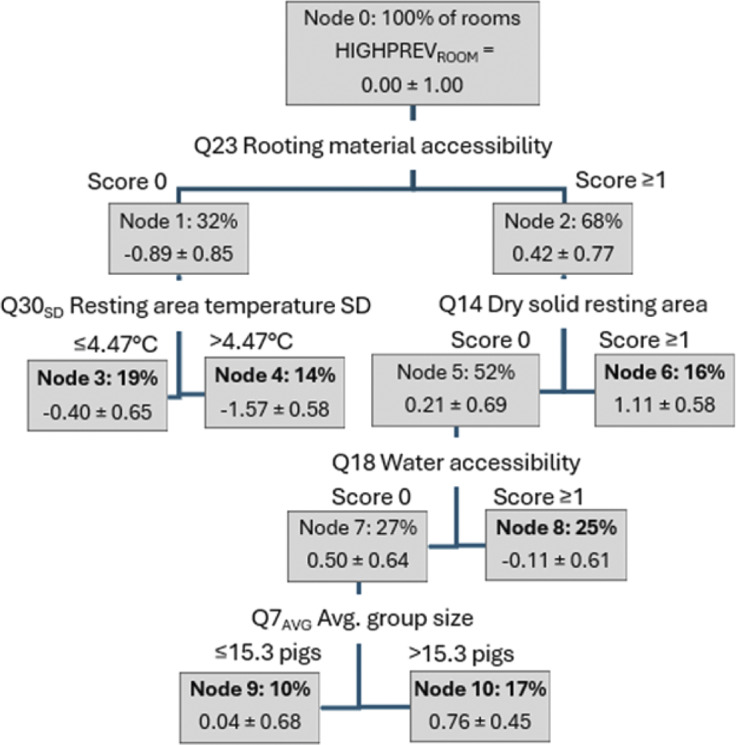
Regression tree diagram predicting the principal component score HIGHPREV_ROOM_, capturing recent signs of tail biting occurring at a high frequency in pens, with a larger score indicating more signs. Each box represents a node and shows the percentage of rooms (n = 81) and mean (± SD) value. HIGHPREV_ROOM_ scores are given for each node. Splitting variables and locations of each split are given along the path (line) graphically representing the split. Terminal nodes (n = 6) are shown in bold.

Component scores are used as outcome variables quantifying tail status in this study. In order to facilitate the understanding of the results from statistical analyses, effects will be referred to as “unfavourable” for an increase in tail damage, and *vice versa.* HIGHPREV_ROOM_ was predicted by a regression tree including five of 59 eligible RQ predictor variables, explaining 69% of total variance. Predicted values correlated highly with HIGHPREV_ROOM_ scores (r_s_ = 0.80; *P* < 0.001). The tree is pictured in Figure 4. Interpretation of the results given in the figure is facilitated by the score average of 0.00, whereby average node scores below zero indicate that the tail status in the rooms forming that node was better than the population average, and *vice versa.* Splits were generally generated as expected, with the situation associated with a higher risk for tail biting yielding an unfavourable effect. The RTA produced six terminal nodes representing subgroups in the data, each of which satisfy a unique set of split conditions along the path. The terminal node n range of 8–15 rooms indicates that RTA predictions were based on a sufficient amount of data throughout the tree. Terminal nodes typically included rooms from two consecutive age categories (nodes 4 and 9 included younger, and nodes 3 and 10 older animals), indicating that risk factors were often age dependent. Nodes 6 and 8 included all age categories.

LMR results are summarised in Table 7. Increasing age (*P* ≤ 0.011 for all pair-wise contrasts) and decreasing average resting area temperature exerted unfavourable effects on HIGHPREV_ROOM_. Effects of the categorical variables enrichment object destructibility (Q25) and cleanliness (Q27) were slightly unclear. For Q25, absence of objects (Q25C9) led to the best, and provision of slightly destructible objects to the worst outcome (Q25C2; pair-wise contrast *P* = 0.006). Indestructible objects (Q25C3) were superior to Q25C2 (*P* = 0.004) but did not differ from Q25C9. Dirty enrichment objects in some pens (Q27C1) were significantly more favourable as compared to clean objects in all pens (Q27C0; *P* = 0.015).

**Table 7.** Effects (*P*-values) of risk factors on latent patterns of occurrence of tail damage on room level (n = 81), and on types of individual-level (n = 777) tail damage in weaner pigs on 10 farms

Predictor	“Recent high-prevalence tb” (HIGHPREV_ROOM_, n = 81)	“Underlying tb” (UNDERLYING_ROOM_, n = 78)	“Recent low-prevalence tb” (LOWPREV_ROOM_, n = 81)	“Relatively fresh tail injury”, (FRESH_IND_, n = 777)	“Acute severe injury” (SEVERE_IND_, n = 776)
Farm ID	N/A	N/A	< 0.001	N/A	N/A
Age category	< 0.001	ns	> 0.10	0.016	> 0.10
UNDERLYING_ROOM_ factor score	N/A	N/A	N/A		0.043
Q4 Room capacity			0.020		
Q7_avg_ Group size					< 0.001
Q8_ind_ Space allowance		0.009			
Q13_ind_ Appropriate resting area				0.009	
Q20 Resources, disadvantaged individuals		0.016			
Q25 Destructibility, objects	0.014				
Q27 Cleanliness, objects	0.034				
Q30_avg_ Lying area temperature	0.033				
Q34 Draught				< 0.001	
Q44 Availability of feed		0.010			
Age category * Q44		0.007			
Q51 Sneezing					0.021
Random effect(s)	Farm	Farm		Farm, pen within farm	Room, pen within room

tb = tail biting. N/A = not eligible as predictor in model. ns: 0.050 ≤ *P* < 0.10. Q = Question in the Risk Factor Questionnaire. *P* > 0.10: included in the model despite of non-significance.

#### LOWPREV_ROOM_ and UNDERLYING_ROOM_

RTA was not able to build satisfactorily performing trees predicting LOWPREV_ROOM_ or UNDERLYING_ROOM_. In the LMR model on LOWPREV_ROOM_, farm was included as a fixed factor as the model failed to converge with the variable as a random effect. Tail health in terms of LOWPREV_ROOM_ was predicted to deteriorate with an increasing number of pigs in the room (Table 7).

**Figure 5.**
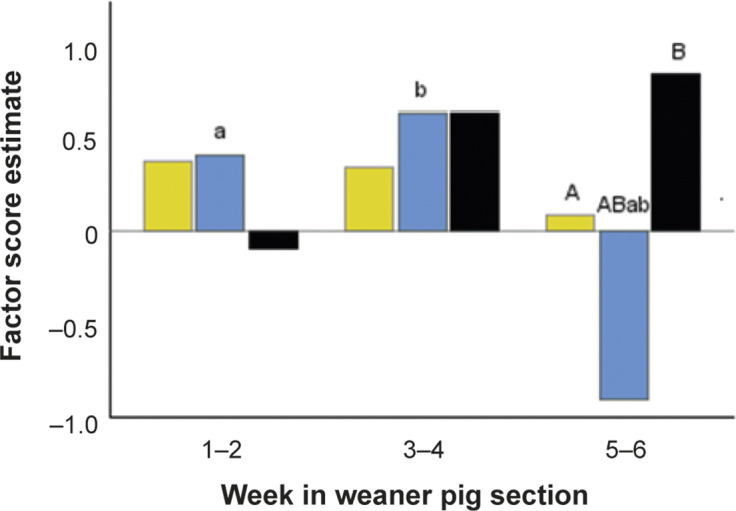
Effects of Q44 “Availability of feed” and age category on the average estimate of a principal component score quantifying signs of underlying tail biting (UNDERLYING_ROOM_) in 81 weaner pig rooms on ten farms. Score 1 represents the worst and –1 the best situation in the data. Q44 categories describe the possibility for all pigs to be able to eat simultaneously and availability of feed: Q44C0 simultaneous/continuous (yellow bar); Q44C1 simultaneous/not continuous (blue bar); Q44C2 not simultaneous/not continuous (black bar). Equal subscripts indicate significant results (*P* ≤ 0.040) in pair-wise comparisons of the interaction between Q44 and age (week in weaner section): lowercase is used for age effects within Q44 category and uppercase for Q44 category effects within age.

UNDERLYING_ROOM_ was affected by an interaction between availability of feed (Q44) and age category, which is visualised as a graph of predicted scores in Figure 5. According to the interaction, the only Q44 category showing an age effect was Q44C1 “All pigs cannot eat simultaneously, but feed is available continuously”, for which tail health was better at weeks 5–6 as compared to both younger age categories (*P* < 0.003 for both pair-wise comparisons). The interaction also showed that Q44 affected UNDERLYING_ROOM_ only at weeks 5–6, when the score was lower in Q44C1 as compared to both other categories (Q44C0 “All pigs able to eat simultaneously” and Q44C2 “All pigs cannot eat simultaneously, and feed is not available continuously”; see Figure 5 for pair-wise contrasts). Increasing space allowance and better resource access for disadvantaged individuals (Q20) was favourable in terms of UNDERLYING_ROOM_. The effects of the Q20 categories can be pictured as linear, indicating an expected unfavourable effect of access to a diminishing resource. The two most extreme categories differed significantly (Q20C0 and Q20C2; *P* = 0.004), but all other pair-wise contracts did not reach significance (0.05 < *P* < 0.10).

### Effects of risk factors on individual-level tail status in problem pens

RTA did not perform satisfactorily in individual-level data as judged by a 77% or higher proportion of unexplained variance, and low correlations between original and predicted values (Pearson coefficient 0.36–0.56; *P* < 0.001, n = 776 for all comparisons). Only LMR results are thus reported (Table 7).

The FRESH_IND_ type of tail injury was affected by age, availability of appropriate resting area (Q13) and draught (Q34). FRESH_IND_ was more prevalent and/or more severe than other types in age category weeks 3–4 as compared to both weeks 1–2 and 5–6 (*P* = 0.013 and *P* = 0.030, respectively). Q13 was defined as a two-climate area in weeks 1–2, and for older pigs a designated resting area. The most favourable alternative was Q13C1 “for at least 90% of animals” (pair-wise contrast; *P* = 0.015 to Q13C2 “< 90%” and *P* = 0.002 to Q13C3 “< 70%”). The Q13C0 category “all animals” was superior only to Q13C3 (*P* = 0.033). The effect of draught was not clear. The best outcome was for Q34C2 “mild levels in some place(s) in resting area”, for which pair-wise contrasts to all other categories were significant (*P* < 0.001). The worst outcome was for Q34C3 “yes, clearly”, for which pair-wise contrasts were significant with Q34C2 and Q34C0 “no” (*P* < 0.001).

SEVERE_IND_ was unfavourably affected by increasing group size and a decreasing score for the room-level factor UNDERLYING_ROOM_. Sneezing (Q51) affected the score as expected, with a less favourable situation predicted for Q51C3 “in > 50% of pens” as compared to Q51C0 “in no pens” and Q51C2 “in 10–50% of pens” (*P* ≤ 0.02).

## Discussion

### Tail health in relation to age

This study aimed to establish associations between risk factors and both the occurrence pattern and quality of signs indicative of tail biting in cross-sectional data in intact-tailed weaner pigs on ten farms. The occurrence of tail lesions, hanging tails and healed tails were expressed as percentages of high-, low- and zero-prevalence pens in 81 rooms. Signs of tail biting increased with increasing age, especially from the first to the second third of the weaner phase. Although our cross-sectional approach does not allow for conclusions regarding an age effect, previous longitudinal data in intact-tailed weaner pigs in comparable environments have yielded similar results. Håkansson *et al.* (2020) saw increases in both prevalence and severity of tail lesions at 4–5 weeks post-weaning, whereas Gentz *et al.* (2020) reported an increasing prevalence over the first seven weeks.

The multidimensional information collected on signs of tail biting was reduced into principal components, describing latent types of occurrence at the room level, as well as latent types of damage in problem pens. The failure of shortened, healed tails to contribute to room-level components indicates that they occurred independently of the other signs. This finding may be explained by the permanent nature of tail shortening, whereas fresh lesions and tail hanging are expected to be present for shorter time-spans.

Two of the three room-level principal components, HIGHPREV_ROOM_ and LOWPREV_ROOM_, together explaining 57% of variability in the data, were formed by a certain *prevalence* of tail-biting signs, not by the *quality* of signs. This indicates that there was little variation across pens in the number of affected animals, whereas the quality of biting (force and/or recurrence) was more variable. It can thus be hypothesised that HIGHPREV_ROOM_ and LOWPREV_ROOM_ represented different levels of motivation for tail biting. The third room-level component, UNDERLYING_ROOM_, was loaded upon by occurrence of hanging tails only, to indicate incoming non-injurious bites, pain, stress (Noonan *et al.* 1994; Wilder *et al.* 2021) or negative emotional state (Reimert *et al.* 2013). The increase in HIGHPREV_ROOM_ with increasing age appears to represent the well-known escalating nature of tail biting activity (Niemi *et al.* 2021).

### Effects of risk factors on latent room-level patterns of occurrence of tail-biting signs

#### HIGHPREV_ROOM_

According to EFSA (2014), classification and regression tree analysis (CART) is preferable to traditional regression when addressing multifactorial phenomena, such as tail biting. Here, RTA (CART for a continuous outcome) was applied to identify relevant risk factors for all six principal components, however, the method only performed satisfactorily for HIGHPREV_ROOM_. Previously, the performance of CART to establish risk factors for tail lesions has proved to be variable. A prediction error of 13% was reported in a dataset of 60 farms (Scollo *et al.* 2017), 38% in 242-farm data, and 49–51% in subpopulations of 97–119 of the latter dataset (EFSA 2014). Possible reasons for the divergence in CART applicability lie beyond the scope of this study.

LMR and RTA were largely in accordance regarding the type of risks associated with HIGHPREV_ROOM_, that is, shortcomings related to the provision of enrichment as well as the immediate environment. According to RTA results, continuous access to material from *two* different sources (e.g. bedding and straw rack) was associated with a more favourable HIGHPREV_ROOM_; as compared to continuous access but from *one* source only, less optimal access to, or no observed rooting material. Although the location of the split may seem to be at a high level of enrichment provision (two sources versus one), the result is in line with the general recommendation for a combination of different objects and/or substrates for point-source enrichment in pigs, based on a literature review by Bracke *et al.* (2006). The enrichment value of diversity was shown in weaner pigs by Trickett *et al.* (2009), who reported that interaction with different, simultaneously presented objects, is summative as compared to presenting the objects one at the time. On the present farms, two sources of material probably often led to a larger absolute amount than one source, which is expected to increase interaction as well, as found for straw in weaner pigs by Kelly *et al.* (2000).

Risk factors predicting HIGHPREV_ROOM_ according to LMR included destructibility (Q25) and cleanliness (Q27) of toys. Dirty toys in some pens counterintuitively predicted a better outcome as compared to clean toys in all pens, however, the former scenario may indicate greater attractiveness for the objects in question. Absence of toys was, also surprisingly, more favourable than provision of slightly destructible toys. This phenomenon may be caused by resource competition when only scarce enrichment was provided; or it may have been explained by a significant inverse relationship on the study farms between, on one hand, provision and destructibility of toys and, on the other, presence of rootable materials. The Finnish interpretation of relevant legislation requires compensation by objects, if rootable material is not always available (Finnish Food Authority 2019). It is unclear why RQ questions on toys produced a better LMR fit than any of the questions directly describing the availability, amount or quality of enrichment materials. It is possible that rootable materials were often provided in small amounts and thus were not always present in the pens. Thus a single observation, which was the method of RQ data collection, may have missed scarcely used consumable enrichment materials, leaving lack of objects a more reliable measure of material provision.

Characteristics of the resting area predicted HIGHPREV_ROOM_ both in RTA (Q14, Accessibility of solid and dry resting area and Q30_SD_, temperature SD on that area) and LMR (Q30_avg_). Unexpectedly, Q30_SD_ exceeding 4.47°C predicted a better outcome than a smaller SD, perhaps due to the positive correlation between Q30_SD_ and Q30_avg_ in the data. Q30_avg_ was on the highest level of all terminal nodes in Node 4 (29.1°C, min 28.8°C max 29.6°C). Eight of the eleven rooms in Node 4 represented age category weeks 1–2, which is the age benefitting the most from a high temperature due to a high post-weaning heat demand, as reviewed by Ramirez *et al.* (2022). It is unclear why the actual temperature (Q30_avg_) did not cause the split in the RTA, however, this variable had a significant and expected effect on LMR, with a higher value predicting a more favourable outcome. Cold temperatures are a powerful stressor that interacts with food intake and health in weaned pigs, especially in the first weeks, as reviewed by Le Dividich and Herpin (1994). During this time, cold may cause morbidity and agonistic behaviour (Ramirez *et al.* 2022).

Shortcomings regarding the availability of a solid and dry resting area (Q14) appeared to be an important risk factor for tail biting on the present farms. In the RTA, the node with the poorest tail health in terms of HIGHPREV_ROOM_ was formed by rooms where this was possible for 90% or less of animals (Node 6; n = 13 rooms). All age categories were represented in this node, with an emphasis on the oldest animals. Inability to rest on a solid and/or dry area may cause cold stress, as both moisture and slats will increase heat loss (Bruce & Clark 1979). Attempts to force entry into an overcrowded desired area will also cause disturbance in the group.

Group size (Q7) and water accessibility (Q18) caused further splits in the RTA. For Q18, any violation to the recommendation (≥ 2 drinkers per pen, ≥ 1 drinker per ten pigs, and true simultaneous access to all drinkers) produced the *better* outcome as compared to meeting all recommendations in all pens in the room. This result was surprising, especially as Finnish producers rank water availability as the fourth most important preventative measure of tail biting on a list of 20 (Valros *et al.* 2016). The data were explored for confounders that would possibly explain the result, but none were identified.

Average group size produced the terminal node with the second poorest tail status in terms of HIGHPREV_ROOM_ (Node 10). A larger group size caused the less desirable result, as expected, with the cut-off set at 15.33 individuals. An increasing or larger group size has been shown to increase tail lesions in intact-tailed fattening pigs (Holmgren & Lundheim 2004; Kallio *et al.* 2018; Wallgren *et al.* 2019). Possible mechanisms include a higher pathogen pressure and difficulty in accessing resources (Boyle *et al.* 2022).

##### LOWPREV_ROOM_

LOWPREV_ROOM_ captured lower prevalence signs of tail biting. The only RQ item predicting LOWPREV_ROOM_ in the LMR was room size, with an expected unfavourable effect of an increasing number of animals. Drawbacks of a large room size include, for example, high levels of noise, shortage of true quiet time, increased pathogen pressure and difficulty achieving a uniform climate throughout all parts of the room. Farm as fixed effect explained a large part of the variability in these data. This implies that significant farm-specific risk factors existed, which were not included in the RQ. Such factors may include genetics, general attitude on the farm, feed, or tail health on introduction to the weaner room. A Danish study by Håkansson *et al.* (2020) found tail lesions not to be uncommon at weaning and that they predicted further tail lesions during the weaner stage.

##### UNDERLYING_ROOM_

UNDERLYING_ROOM_, that is, prevalence of hanging tails in the absence of lesions, was affected by resource availability in ways that were entirely logical. A smaller space allowance (Q8_ind_) and restricted accessibility of resources for disadvantaged individuals (Q20; typically two drinkers very close to each other) were unfavourable, irrespective of age, whereas the actual availability of feed (Q44) became important in the oldest animals. In the weeks 5–6 cohort, uninterrupted availability of feed (typically from a feeder) was more favourable than restricted (typically from a feeder, but with interruptions), with a similar trend as compared to simultaneous feeding (typically long trough). The latter effect indicated that availability of feed became impaired when the system operated close to its limits in terms of total mass of animals. Any form of restriction to feed availability may cause damaging behaviours in pigs, as reviewed by Boyle *et al.* (2022). Finnish producers rank (enough) feeding space as the most important preventative measure for tail biting (Valros *et al.* 2016). Although a long trough with space for the entire group is considered a low-risk option, and 25 cm per animal was provided on the present farms, the results do indicate that a certain degree of crowding was present in large animals. The observed effects of (decreasing) space allowance is well in line with research showing an association with tail-lesion prevalence in intact-tailed weaner pigs (Grümpel *et al.* 2019), fattening pigs (Munsterhjelm *et al.* 2015) and both (Scollo *et al.* 2017).

The finding that shortcomings in resource access and not the other tail-biting signs, including visible lesions affected UNDERLYING_ROOM_, is surprising. The typical aetiology for sudden, forceful tail biting, as categorised by Taylor *et al.* (2010), is lack of resources. Hanging tails in the absence of injuries in the present animals may indicate that the shortage was not severe enough to cause forceful tail biting. Instead, the animals appear to have experienced stress, negative emotional states and/or milder forms of tail biting (Noonan *et al.* 1994; Reimert *et al.* 2013; Wilder *et al.* 2021).

### Latent types of tail injury in problem pens

Types of tail lesions in problem pens differed greatly between farms. Three distinct types were identified in the pooled data. HEALED_IND_ captured previous severe tail biting, FRESH_IND_ indicated that active and widespread tail biting had been going on for at least a few days, whereas SEVERE_IND_ included grave injuries. As SEVERE_IND_ was negatively loaded by smaller lesions (0.5–2 cm), the damage type closely resembles tail biting of the “sudden forceful” and “obsessive” types, as categorised by Taylor *et al.* (2010). The former is typically caused by resource shortage, whereas the latter has several aetiologies, including obsessive individual biters, acceleration of milder forms of biting, or spontaneous outbreaks (Taylor *et al.* 2010). Shortage of resources was not identified as a risk factor for SEVERE_IND_ in the present data. We thus suggest that tail lesions predominantly were caused by obsessive-type tail biting. Risk factors of a less intensive, but continuous and longer-term nature in group size and sneezing frequency were found to affect SEVERE_IND_. Obsessive tail biting may have developed as an acceleration of less intensive biting, as described by Taylor *et al.* (2010). Respiratory pathology has repeatedly been associated with tail lesions in slaughterhouse material, as reviewed by Boyle *et al.* (2022); however, data in live pigs, especially weaner pigs, are scarce. The observed preventative effect of UNDERLYING_ROOM_, showing that *less* pens in the room with hanging tails as the worst sign of tail biting increased the relative importance of SEVERE_IND_ in problem pens, is difficult to explain. If a lower UNDERLYING_ROOM_ score meant more actual tail lesions in the room, the effect would capture the escalation or an outbreak in the room, however, this is entirely speculative.

FRESH_IND_ was negatively loaded by scarred lesions, indicating that pens with active and widespread tail biting had either not experienced previous injurious biting, or that scarred tails had recently been targeted again. FRESH_IND_ could thus capture a severe outbreak, a continuum of a longer lasting problem, or both. Associated risk factors included lack of comfortable resting areas, draughts and a lack of roofed space. Draughts are an acknowledged risk in the cool Finnish climate and is high on the list of the most important preventative measures for tail biting, according to producers (Valros *et al.* 2016). Draughts are aversive and may cause agonistic behaviour and morbidity in weaner pigs (Scheepens *et al.* 1991a,b).

The importance of HEALED_IND_ did not increase with age, although a lower prevalence of healed and shortened tails could have been expected in the first as compared to later thirds. Healed and shortened tails were present in 5.1%, 4.4% and 6.6% of individually assessed animals in age categories weeks 1–2, 3–4 and 5–6. Complete healing of a traumatic tail amputation in two weeks or less appears unlikely, as healing times of 4–8 weeks have been reported after tail docking (Sandercock *et al.* 2016). It is thus likely that some animals entered the weaner pig section with shortened tails.

### Animal welfare implications

The use of tail (biting) lesions, an indicator of growing pig welfare, as the outcome in this study allows for robust interpretations of the results in terms of animal welfare. The rapid increase in tail lesions in the first weeks post-weaning suggests that the animals experienced significant stress, the sources of which were at least partly identified as shortcomings in environment and husbandry. Correction of these factors would have a profound effect on pig welfare not only immediately, but also in the following weeks and months, if the decreased incidence of tail wounds and their complications lead to a lowered threshold for tail biting in the population.

## Conclusion

The general increase in indicators indicative of tail biting over the weaner stage, especially from the first to the second third, implies that important causative factors exist in the time around weaning. Solving the problems in this phase could decrease tail biting throughout the entire pig production chain, as animals have less previous experience of the behaviour. The identified risk factors emphasise basic, well-known needs for weaner pigs with adequate resting areas and enrichment as the most important shortcomings on the present farms. Different risk factors could be associated with different types of tail lesions in problem pens, suggesting distinct aetiologies.

## Supporting information

10.1017/awf.2026.10076.sm001 Munsterhjelm et al. supplementary material Munsterhjelm et al. supplementary material

## Supplementary material

The supplementary material for this article can be found at http://doi.org/10.1017/awf.2026.10076.
