# Supplementary material for: Risk factors for signs of tail biting in intact-tailed weaner pigs: A cross-sectional study
Source: Anim Welf. 2026 Mar 27;35:e22. doi: 10.1017/awf.2026.10076 (PMC13101024; doi:10.1017/awf.2026.10076)
Supplement: Munsterhjelm et al. supplementary material [file S0962728626100761sup001.pdf]

Risk factors for signs of tail biting in intact-tailed weaner pigs: A cross-sectional study: Supplementary material

Camilla Munsterhjelmhttps://orcid.org/0000-0003-2949-2064, Miina Tuominen-Brinkas, Hikka Koskikallio, Mari Heinonen, Kristina Ahlqvist, Anna Valroshttps://orcid.org/0000-0002-4431-3346

Research Centre for Animal Welfare, Department of Production Animal Medicine, Koetilantie 7, 00014 University of Helsinki, Finland

Author for correspondence: Camilla Munsterhjelm, email: camilla.munsterhjelm@helsinki.fi

Tables S1a-c. Tail biting risk factor assessment in 32 weaner pig rooms on 10 farms.

Table S1a. Risk Factor Questionnaire. Answer categories are specified in the shaded columns.

Area/ subarea	Question no (Q)	Assess-ment order	Question	Specifications	Unit or variable in analyses	Category 0	Category 1	Category 2	Category 3	Category 9
Characteri stics/ farm	**1**	N/A	**Weaner pig capacity**	Average number of weaner pigs on the farm during 12 months	Number					
Characteri stics/ animals	**2**	N/A	**Age category**	Calculated from the day of arrival; or the middle day in rooms with two arrival days.	Category	1-2 week in weaner pig section	3-4 week in weaner pig section	5-6 week in weaner pig section		
Characteri stics/ animals	**3**	38	**Weight**	Estimated typical weight in room	Category	< 10 kg	10-20 kg	>20 kg		
Resources / room	**4**	32	**Room capacity**	Number of pigs in room	Number					
Resources / room	**5**	32	**Sick pens**	Relative number of sick pens in room	% of pens					
Resources / pen	**6**	33	**Pen size equality**	Are all pens of equal size in the room?	Category	Yes	No			
Resources / pen	**7**	4	**Group size**	Number of pigs in pen	Average ($Q7_{avg}$), st.dev. ($Q7_{SD}$), $Q7_{min}$, $Q7_{max}$ in room					
Resources / pen	**8**	34	**Space allowance**	Pen area; one measurement per typical pen in room	Average area per pen in room ($Q8_{pen}$); average area per pig in room ($Q8_{ind}$)					
Resources / pen	**9**	36	**Solid floor area**	Available area; one measurement per typical pen in room	Average area per pen in room ($Q9_{pen}$); average area per pig in room ($Q9_{ind}$)					

Area/ subarea	Question no (Q)	Assess-ment order	Question	Specifications	Unit or variable in analyses	Category 0	Category 1	Category 2	Category 3	Category 9
Resources / pen	**10**	39	**Non-solid floor**	Available slatted or perforated area; one measurement per typical pen in room	The most representative category	Less than 1/3 of floor area	1/3–1/2 of floor area	>½ of floor area		
Resources / pen	**11**	40	**Activity areas**	There are distinct and functioning activity areas including feeding, resting and dunging areas. Active pigs do not bother resting pigs.	The most representative category	Yes	Yes, almost	Requirements are fulfilled to some degree (e.g. 2 diff. activity areas)	Require-ments are not fulfilled	
Resources / resting area	**12**	2	**Resting location**	Part of resting pigs on the intended area of resting	The most representative category	All pigs	Most pigs	About half	Less than half	Not enough resting pigs
Resources / resting area	**13**	43	**Availability of appropriate resting area**	Can all pigs rest comfortably and simultaneously 1) under the two-climate roof for the first two weeks and 2) on the resting area thereafter (observe or evaluate)?	The most representative category	Both requirements fulfilled	Both require-ments true for at least 90% of pigs	Both require-ments true for 70-89% of pigs	Other	
Resources / resting area	**14**	44	**Accessibility of solid and dry resting area**	Part or group able to rest simultaneously on solid, dry area	The most representative category	All pigs	>90% of pigs	50-90%	<50%	
Resources / feed and water	**15**	37	**Feeding space**	Total length of trough or feeding space of feeder; one measurement per typical pen in room	Average length per pen in room ($Q15_{pen}$); average length per pig in room ($Q15_{ind}$)					
Resources / feed and water	**16**	11	**Simultaneous feeding**	Percentage of animals able to eat simultaneously: observe feeding or evaluate based on a requirement of 15 (small) -25 cm (large weaner pigs) feeding space/ pig	The most representative category	>90%	50-90%	<50%-10%	<10%	
Resources / feed and water	**17**	47	**Water nipple placement**	Are there nipples in less desirable locations (resting area, corner, other location difficult to reach)?	The most representative category	No	Not in resting area but in other undesirable location	Some (not all) nipples are in the resting area	All nipples are in the resting area	

Area/ subarea	Question no (Q)	Assess-ment order	Question	Specifications	Unit or variable in analyses	Category 0	Category 1	Category 2	Category 3	Category 9
Resources / feed and water	**18**	46	**Water accessi-bility**	1) at least one nipple per 10 pigs; 2) at least 2 nipples can be used simultaneously pen.	The most representative category	Both requirements fulfilled in all pens	All nipples cannot be accessed simultaneously (e.g. close to each other)	One require-ment is fulfilled	None of the require-ments are fulfilled	
Resources / feed and water	**19**	15	**Water flow**	3 assessments in different pens in the room	Average ($Q19_{avg}$), st.dev. ($Q19_{SD}$), $Q19_{min}$, $Q19_{max}$ in room					
Resources / feed and water	**20**	42	**Resource accessibility for unprivileged individuals**	Are other individuals in the way if an individual wants to access food or water?	The most representative category	No	Possibly, sometimes	Yes, clearly, often		
Resources / feed and water	**21**	9	**Resource accessibility**	Crowding or queuing at feeder or water nipple	Category	Not observed	observed in 1-2 pens	In ≥3 pens	In most pens	
Resources / enrich-ment	**22**	53	**Enrichment accessibility**	Part of pigs that can use enrichment (any form: bedding, roughage, toys etc) simultaneously if desired.	The most representative category	All pigs. If only toys are present: 1) ≥ one per 2 pigs; 2) placed at different places in pen.	Not all but ≥ 50%. Enrichment is available at >1 place in pen. If only toys are present: ≥ one per 5 pigs.	<50% of pigs or all enrich-ment in one place in the pen	No enrich-ment observed	
Resources / enrich-ment	**23**	48	**Rooting material accessiblity**	Do the animals have continuous access to rooting material (roughage or any substrate that can form a pile on the floor)? A source is one type of material, but the same material on the floor and in a rack is two sources.	The most representative category	Yes, from two sources (e.g. bedding and straw rack)	Yes, from one source (e.g. bedding or straw rack)	Yes, but access is limited due to e.g. small spaces in straw rack	Access is not continu-ous	
Resources / enrich-ment	**24**	49	**Enrichment quality of rooting material**		The most representative category	Straw, hay or similar	Paper or pressed (not powdery) peat or similar	Sawdust, wood shavings or non-pressed (powdery) peat or similar	No rooting material observed	

Area/ subarea	Question no (Q)	Assess-ment order	Question	Specifications	Unit or variable in analyses	Category 0	Category 1	Category 2	Category 3	Category 9
Resources / enrich-ment	**25**	51	**Quality of toys**	Deformability refers to the possibility to dinge, alter the shape or destruct the object	The most representative category	Readily deformable (e.g. wood, rope) and pigs are observed using the toys	Readily deformable	Slightly deformable (soft plastic or similar)	Not deformable (hard plastic or metal)	
Resources / enrich-ment	**26**	54	**Bedding or enrichment material quality**	Quality is assessed in terms of dirtiness, dust, mold, decomposition etc	The most representative category	No material is of poor quality	The material is dusty, otherwise ok	Some material is of poor quality	Most of the material is of poor quality	Not enough material to assess
Resources / enrich-ment	**27**	52	**Cleanliness of toys**	Dirty means visible material on the object	Category	Toys are clean in all pens	Toys are clean in most pens	Toys are dirty in some pens	Toys are dirty in most pens	No toys in pen
Climate	**28**	50	**Bedding coverage**	Part of floor *not* covered by bedding	The most representative category	<20%	20–50%	>50-80%	>80% or no bedding	
Climate	**29**	35	**Roofed area**	Roof refers to two-climate system; one measurement per typical pen in room	Average area per pen in room ($Q29_{pen}$); average area per pig in room ($Q29_{ind}$)					
Climate	**30**	3	**Resting area temperature**	Laser device measurement in the middle of the resting area in 3 different pens	Average ($Q30_{avg}$), st.dev. ($Q30_{SD}$), $Q30_{min}$, $Q30_{max}$ in room					
Climate	**31**	1	**Formation of resting pigs in pen**		The most representative category	As one distinct group, pigs are not on top of each other	As >1 distinct groups, not on top of each other or spread out in the pen	Piling: very close together, some on top of each other	Separated: far from each other, spread out in the pen	Not enough resting pigs
Climate	**32**	5	**Panting**	All occurrences in the room during the assessment are counted	Category	Not observed	<5% of pigs in room	5-10% of pigs in room	>10% of pigs in room	
Climate	**33**	6	**Shivering**	All occurrences in the room during the assessment are counted	Category	Not observed	<5% of pigs in room	5-10% of pigs in room	>10% of pigs in room	

Area/ subarea	Question no (Q)	Assess-ment order	Question	Specifications	Unit or variable in analyses	Category 0	Category 1	Category 2	Category 3	Category 9
Climate	**34**	41	**Draught**	As perceived on wet hand at pig height in pen	The most representative category	Not perceived	Mild levels at some place(s) in resting area	Mild levels throughout the pen	Yes, clearly	
Climate	**35**	31	**Mist system**	A system enabling spraying the dunging area with a fine mist of water is installed and working	Category	Available in all pens	Available in some pens	No		
Climate	**36**	28	**Air dryness**	As perceived by assessor	Category	Not perceived	A little	Clearly		
Climate	**37**	27	**Air humidity**	As perceived by assessor	Category	Not perceived	A little	Humidity is present on walls		
Climate	**38**	26	**Ammonia**	As perceived by assessor	Category	Not perceived	Perceived at mild levels at some places in room (not all the time)	Perceived at mild levels throughout room	Clearly	
Climate	**39**	29	**Noise**	Is the noise level exceeding 65 dB ("speaking loud") at pig level in pens?	Category	Not perceived	At some point but not continuously, e.g. due to running of a machine	Continuously in some pens	Continuousl y throughout the room	
Climate	**40**	30	**Lightning**		Category	Excellent; could read newspaper almost anywhere in room	Good; could read newspaper in most places in the room	Very variable lightning in room	Poor lightning in room	
Resources / feed	**41**	12	**Feed type**		The most representative category	Liquid	Dry, pelleted	Other type		
Resources / feed	**42**	13	**Feed quality**	Assess visible feed in the trough or feeder. Good quality: fresh and clean.	Category	Good	Good in almost all pens	Other		Not enough feed visible for assess-ment
Resources / feed accessibili ty	**43**	14	**Feeding place cleanliness**	Any material other than fresh food is considered dirt	Category	Less than 10% of feeders/ troughs dirty	10-50% of feeders/ troughs dirty	>50-90% of feeders/ troughs dirty	>90% of feeders/ troughs dirty	

Area/ subarea	Question no (Q)	Assess-ment order	Question	Specifications	Unit or variable in analyses	Category 0	Category 1	Category 2	Category 3	Category 9
Resources / feed accessibility	**44**	10	**Availability of feed**	Observe or evaluate based on a requirement of 15-25 cm (small-large weaner pigs) feeding space/ pig	The most representative category	All pigs able to eat simultaneously	All pigs cannot eat simultaneously, but feed is available continuously	All pigs cannot eat simultaneous-ly, and feed is not available continuously		
Resources / feed accessibility	**45**	8	**Changing of feeding place**	Changing of feeding place in pigs currently eating	The most representative category	Not observed	observed 1-2 times in a pen	≥3 times in a pen	most animals in pen change places	Not enough animals eating
Resources / feed accessibility	**46**	17	**Pigs with empty stomach**		Category	Not observed	<5% of pigs in room	5-10% of pigs in room	>10% of pigs in room	
Health and hygiene	**47**	16	**Water nipple cleanliness**	Dirtiness of water nipples	Category	Less than 10% of nipples dirty	10-50% of nipples dirty	>50-90% of nipples dirty	>90% of nipples dirty	
Health and hygiene	**48**	45	**Pen cleanliness**	Location of dung: dunging area is the only desirable location	The most representative category	Observed only in dunging area	Some dung both in resting area and in another undesirable location of pen	Dung at many places in the pen		
Health and hygiene	**49**	23	**Diarrhoea**	Any observation of liquid feces	Category	Not observed	<10% of pens	5-10% of pens	5-10% of pens	
Health and hygiene	**50**	18	**Coughing**	At least one observation	Category	Not observed	<10% of pens	10-50% of pens	>50% of pens	
Health and hygiene	**51**	19	**Sneezing**	At least one observation	Category	Not observed	<10% of pens	10-50% of pens	>50% of pens	
Health and hygiene	**52**	24	**Scratching**	All occurrences in the room during the observation are counted	Category	Not observed	<5% of pigs in room	5-10% of pigs in room	>10% of pigs in room	
Health and hygiene	**53**	22	**Pale pigs**	Visibly pale skin colour	Category	Not observed	<5% of pigs in room	5-10% of pigs in room	>10% of pigs in room	
Health and hygiene	**54**	21	**Runts**	All underdeveloped individuals in room are counted	Category	Not observed	<5% of pigs in room	5-10% of pigs in room	>10% of pigs in room	
Health and hygiene	**55**	20	**Size heterogeneity**	At least one individual of significantly different size in the pen	Category	Not observed	<10% of pens	10-50% of pens	>50% of pens	

Area/ subarea	Question no (Q)	Assess-ment order	Question	Specifications	Unit or variable in analyses	Category 0	Category 1	Category 2	Category 3	Category 9
Behaviour	**56**	25	**Fighting lesions**	At least 5 scratches or lesions indicative of fighting on one side of the body	% of pens in room with at least one case					
Behaviour	**57**	7	**Restlessness**	All occurrences of rapid tail wagging, fighting or similar during the observation are counted	Category	Not observed	<5% of pigs in room	5-10% of pigs in room	>10% of pigs in room	

Table S1b. Risk Factor Questionnaire results. Percentage of rooms (n=32) in the different categories of categorical variables.

Question no (Q)	Question	Answer category				
		0	1	2	3	9
3	Weight	26.7 %	63.3 %	10.0 %		
6	Pen size equality	93.5 %	6.5 %			
10	Non-solid floor	26.7%	63.3%	10.0%		
11	Activity areas	77.4 %	22.6 %	0.0 %	0.0 %	
12	Resting location	58.6 %	41.4 %	0.0 %	0.0 %	0.0 %
13	Availability of appropriate resting area	61.3 %	16.1 %	9.7 %	12.9 %	
14	Accessibility of solid and dry resting area	77.4 %	16.1 %	0.0 %	6.5 %	
16	Simultaneous feeding	29.0 %	25.8 %	38.7 %	0.0 %	6.5 %
17	Water nipple placement	100.0 %	0.0 %	0.0 %		
18	Water accessibility	22.6 %	41.9 %	16.1 %	19.4 %	
20	Resource accessibility for unprivileged individuals	51.6 %	32.3 %	16.1 %		
21	Resource accessibility	71.0 %	16.1 %	0.0 %	9.7 %	
22	Enrichment accessibility	0.0 %	0.0 %	0.0 %	100.0 %	
23	Rooting material accessibility	32.3 %	25.8 %	6.5 %	35.5 %	
24	Rooting material quality	38.7 %	3.2 %	51.6 %	6.5 %	
25	Quality of toys	0.0 %	6.5 %	32.3 %	45.2 %	16.1 %
26	Bedding or enrichment material quality	92.9 %	7.1 %			
27	Cleanliness of toys	48.4 %	29.0 %	0.0 %	6.5 %	16.1 %
28	Bedding coverage	0.0 %	41.9 %	22.6 %	35.5 %	
31	Formation of resting pigs	51.6 %	29.0 %	3.2 %	0.0 %	16.1 %
32	Panting	100.0 %	0.0 %	0.0 %		
33	Shivering	100.0 %	0.0 %	0.0 %		
34	Draught	80.6 %	9.7 %	6.5 %	3.2 %	
35	Mist system	0.0 %	0.0 %	100.0 %		
36	Air dryness	48.4 %	48.4 %	3.2 %		

37	Air humidity	93.5 %	6.5 %	0.0 %		
38	Ammonia	51.6 %	38.7 %	3.2 %	6.5 %	
39	Noise	83.9 %	9.7 %	3.2 %	3.2 %	
40	Lightning	77.4 %	6.5 %	16.1 %	0.0 %	
41	Feed type	71.0 %	29.0 %	0.0 %		
42	Feed quality	83.9 %	9.7 %	6.5 %		0.0 %
43	Feeding place cleanliness	77.4 %	19.4 %	3.2 %	0.0 %	
44	Availability of feed	45.2 %	32.3 %	22.6 %		
45	Changing of feeding place	16.7 %	50.0 %	33.3 %	0.0 %	
46	Pigs with empty stomach	27.6 %	58.6 %	10.3 %	3.4 %	0.0 %
47	Water nipple cleanliness	90.3 %	0.0 %	0.0 %	9.7 %	
48	Pen cleanliness	58.1 %	35.5 %	6.4 %		
49	Diarrhoea	35.5 %	25.8 %	22.6 %	16.1 %	
50	Coughing	77.4 %	22.6 %	0.0 %	0.0 %	
51	Sneezing	22.6 %	6.5 %	48.4 %	22.6 %	
52	Scratching	96.8 %	3.2 %	0.0 %	0.0 %	
53	Pale pigs	87.1 %	12.9 %	0.0 %	0.0 %	
54	Runts	6.5 %	74.2 %	16.1 %	3.2 %	
55	Size heterogeneity	30.0 %	10.0 %	26.7 %	33.3 %	
57	Restlessness	33.3 %	40.0 %	23.3 %	0.0 %	

Table S1c. Risk Factor Questionnaire results for continuous variables.

Question no (Q)	Question	Unit	Week 1-2, n=9 rooms			Week 3-4, n=13 rooms			Week 5-6, n=9 rooms		
			Avg	Min	Max	Avg	Min	Max	Avg	Min	Max
4	Room capacity	pigs per room	348	120	960	323	156	644	296	108	480
5	Sick pens	% of pens	4,3%	0,0%	12,5%	4,1%	0,0%	15,4%	4,5%	0,0%	12,5%
7	Group size	n pigs	20	12	27	18.5	12	33	19.5	13	27
8	Space allowance	m^2 per pen	8.6	5.4	11.5	8.8	5.4	15.3	8.8	5.4	11.5
		m^2 per pig	0.44	0.37	0.57	0.48	0.38	0.54	0.46	0.33	0.6
9	Solid floor area	m^2 per pen	5.6	3.1	7.9	5.4	2.6	9.3	6.0	3.6	7.9
		m^2 per pig	0.28	0.17	0.35	0.29	0.14	0.36	0.32	0.22	0.52
15	Feeding space	cm per pen	250	70	490	242	70	490	308	70	490
		cm per pig	14	3	27	15	3	26	17	3	25
19	Water flow	l average	1.7	0.8	4.0	1.4	0.7	4.8	1.9	0.8	4.2
		l st. dev.	0.2	0.0	0.7	0.3	0.0	0.8	0.4	0.0	1.2
		l min	1.6	0.7	4.0	1.2	0.4	4.0	1.6	0.7	3.8
		l max	2.0	0.8	4.0	1.7	0.8	5.6	2.5	0.9	4.8
29	Roofed area	m^2 per pen	2.1	1.3	3.0	2.0	0.0	3.0	1.9	0.0	3.0
		m^2 per pig	0.1	0.1	0.2	0.1	0.1	0.2	0.1	0.0	0.2
30	Resting area temperature	◦C average	27.1	22.6	30.6	25.5	19.0	30.9	23.8	18.8	28.3
		◦C st. dev.	3.9	0.6	6.7	2.8	0.2	5.7	2.1	0.5	3.8
		◦C min	22	18	26	22	19	29	22	17	27
		◦C max	30	23	34	28	21	34	26	20	31
56	Fighting lesions	in % of pens	2.8 %	0.0 %	25.0 %	4.1 %	0.0 %	17.9 %	11.0 %	0.0 %	62.5 %

Table S2. Prevalence of tail biting signs in 81 rooms of weaner pigs on 10 farms. Percentages refer to the relative number of pens in the room within each variable. Data are given as average (min-max, if present). A "lesion" is at least reddening indicative of biting, evident without touching the tail.

Farm	Lesions in > 10%[a]		Lesions in ≤ 10%[ab]		Hanging tail in > 10%[a]		Hanging tail in ≤ 10%[ab]		No acute signs[c]		Healed, shortened tail in > 10%[a]	
1	33.3%	(12.5-62.5)	4.2%	(0.0-6.3)	16.7%	(12.5-18.8)	2.1%	(0.0-6.3)	43.8%	(12.5-62.5)	0.0%	
2	48.7%	(10.0-90.0)	11.8%	(0.0-20.0)	23.6%	(10-30.8)	0.0%		15.9%	(0.0-40.0)	31.0%	(10.0-60.0)
3	32.8%	(0.0-83.3)	0.0%		37.2%	(16.7-65.0)	25.0%	(0.0-50.0)	5.0%	(0.0-10.0)	0.0%	
4	2.8%	(0.0-8.3)	0.0%		27.8%	(0.0-58.3)	16.7%	(8.3-25.0)	52.8%	(33.3-83.3)	0.0%	
5	49.7%	(7.7-91.7)	3.8%	(0.0-7.7)	27.2%	(8.3-46.2)	11.5%	(0.0-23.1)	7.7%	(0.0-15.4)	84.0%	(83.3-84.6)
6	1.7%	(0.0-6.7)	6.2%	(0.0-25.0)	20.0%	(0.0-66.7)	15.8%	(0.0-50.0)	56.3%	(25-93.3)	0.0%	
7	38.9%	(33.3-50)	13.9%	(8.3-16.7)	13.9%	(0.0-25.0)	11.1%	(8.3-16.7)	22.2%	(0.0-50.0)	5.6%	(0.0-16.7)
8	21.7%	(10.0-45.0)	11.7%	(5.0-20.0)	18.3%	(10.0-35.0)	16.7%	(10.0-25.0)	31.7%	(0.0-50.0)	0.0%	
9	23.1%	(15.4-30.8)	17.3%	(11.5-23.1)	19.2%	(7.7-30.8)	17.3%	(11.5-23.1)	23.1%	(19.2-26.9)	1.9%	(0.0-3.8)
10	18.8%	(0.0-31.3)	8.8%	(0.0-12.5)	17.5%	(0.0-31.3)	10.0%	(6.3-12.5)	45.0%	(18.8-93.8)	3.8%	(0.0-12.5)

[a]The percentage refers to individuals in a pen [b]At least one finding in the pen is required, but the total amount is ≤ 10% of animals. [c]Tail lesions or hanging tails.

Table S3. Prevalence of tail biting signs in altogether 777 weaner pig in 35 pens with high tail lesion prevalence on 10 farms.

	Tail shortening			Tail lesion type[1]				Lesion freshness[2]		
Farm	No	Shortened	Stump	No	< 5 mm	0.5-2 cm	> 2 cm	Fresh	Crust	Healed
1	39.7 %	51.5 %	8.8 %	23.5 %	5.9 %	25.0 %	45.6 %	62.3 %	35.8 %	1.9 %
2	8.0 %	51.8 %	40.1 %	5.8 %	1.5 %	22.6 %	70.1 %	82.0 %	15.0 %	3.0 %
3	55.1 %	40.7 %	4.2 %	18.6 %	2.5 %	38.1 %	40.7 %	69.1 %	29.9 %	1.0 %
4	36.4 %	63.6 %	0.0 %	9.1 %	27.3 %	27.3 %	36.4 %	80.0 %	20.0 %	0.0 %
5	47.3 %	52.7 %	0.0 %	42.9 %	0.0 %	35.2 %	22.0 %	36.2 %	37.7 %	26.1 %
6	93.8 %	6.2 %	0.0 %	61.7 %	1.2 %	19.8 %	17.3 %	41.9 %	58.1 %	0.0 %
7	44.7 %	42.1 %	13.2 %	5.3 %	0.0 %	6.6 %	88.2 %	93.2 %	5.5 %	1.4 %
8	38.7 %	59.7 %	1.6 %	25.8 %	1.6 %	27.4 %	45.2 %	72.9 %	22.9 %	4.2 %
9	10.7 %	72.6 %	16.7 %	9.5 %	1.2 %	21.4 %	67.9 %	94.7 %	5.3 %	0.0 %
10	20.4 %	75.5 %	4.1 %	36.7 %	0.0 %	38.8 %	24.5 %	50.0 %	27.5 %	22.5 %

[1]Only skin-penetrating lesions. [2]Percentage of tails with lesions. [2]More than one freshness type is recorded if present on a tail.
